# Albumin Levels and Risk of Early Cardiovascular Complications After Ischemic Stroke: A Propensity-Matched Analysis of a Global Federated Health Network

**DOI:** 10.1161/STROKEAHA.123.044248

**Published:** 2024-02-07

**Authors:** Tommaso Bucci, Daniele Pastori, Pasquale Pignatelli, George Ntaios, Azmil H. Abdul-Rahim, Francesco Violi, Gregory Y.H. Lip

**Affiliations:** 1 Liverpool Centre for Cardiovascular Science at University of Liverpool, Liverpool John Moores University and Liverpool and Heart and Chest Hospital, United Kingdom (T.B., D.P., A.H.A.-R., G.Y.H.L.).; 2 Department of General and Specialized Surgery, Sapienza University of Rome, Italy (T.B.).; 3 Department of Clinical Internal, Anesthesiologic and Cardiovascular Sciences, Sapienza University of Rome, Italy (D.P., P.P., F.V.).; 4 Department of Internal Medicine, Faculty of Medicine, School of Health Sciences, University of Thessaly, Larissa, Greece (G.N.).; 5 Stroke Division, Department of Medicine for Older People, Whiston Hospital, St Helens and Knowsley Teaching Hospitals NHS Trust, Prescot, United Kingdom (A.H.A.-R.).; 6 Danish Center for Clinical Health Services Research, Department of Clinical Medicine, Aalborg University, Denmark (G.Y.H.L.).

**Keywords:** albumins, ischemic stroke, mortality, myocardial infarction, oxidative stress, thrombosis

## Abstract

**BACKGROUND:**

No studies have investigated the association between albumin levels and the risk of early cardiovascular complications in patients with ischemic stroke.

**METHODS:**

Retrospective analysis with a federated research network (TriNetX) based on electronic medical records (*International Classification of Diseases-Tenth Revision-Clinical Modification* and logical observation identifiers names and codes) mainly reported between 2000 and 2023, from 80 health care organizations in the United States. Based on albumin levels measured at admission to the hospital, patients with ischemic stroke were categorized into 2 groups: (1) reduced (≤3.4 g/dL) and (2) normal (≥3.5 g/dL) albumin levels. The primary outcome was a composite of all-cause death, heart failure, atrial fibrillation, ventricular arrhythmias, myocardial infarction, and Takotsubo cardiomyopathy 30 days from the stroke. Secondary outcomes were the risk for each component of the primary outcome. Cox regression analyses were used to calculate hazard ratios (HRs) and 95% CIs following propensity score matching.

**RESULTS:**

Overall, 320 111 patients with stroke had normal albumin levels (70.9±14.7 years; 48.9% females) and 183 729 (57.4%) had reduced albumin levels (72.9±14.3 years; 50.3% females). After propensity score matching, the primary outcomes occurred in 36.0% of patients with reduced and 26.1% with normal albumin levels (HR, 1.48 [95% CI, 1.46–1.50]). The higher risk in patients with reduced albumin levels was consistent also for all-cause death (HR, 2.77 [95% CI, 2.70–2.84]), heart failure (HR, 1.31 [95% CI, 1.29–1.34]), atrial fibrillation (HR, 1.11 [95% CI, 1.09–1.13]), ventricular arrhythmias (HR, 1.38 [95% CI, 1.30–1.46]), myocardial infarction (HR, 1.60 [95% CI, 1.54–1.65]), and Takotsubo cardiomyopathy (HR, 1.51 [95% CI, 1.26–1.82]). The association between albumin levels and the risk of cardiovascular events was independent of advanced age, sex, multimorbidity, and other causes of hypoalbuminemia. A progressively increased risk of adverse events was found in patients with mild and severe reduced compared to normal albumin levels.

**CONCLUSIONS:**

Albumin levels are associated with the risk of early cardiovascular events and death in patients with ischemic stroke. The potential pathophysiological or therapeutic roles of albumin in patients with stroke warrant further investigation.

Almost 25% of patients with ischemic stroke develop early cardiovascular complications with significantly increased risk of morbidity and mortality.^1^ Following poststroke neuronal injury, the release of large amount of catecholamines and cytokines induces a systemic inflammatory response that could lead to myocardial dysfunction, thrombosis, and arrhythmias.^2^ A wide range of cardiac complications can follow in the 30 days of an ischemic stroke, with the possible onset of acute coronary syndrome, heart failure, atrial fibrillation, ventricular arrhythmias, and Takotsubo cardiomyopathy, conferring the so-called Stroke Heart Syndrome.^3^

Previous studies showed that inflammation plays a pivotal role in inducing these manifestations and that C-reactive protein levels correlate with the cerebral infarct volume and the risk of early cardiovascular events.^4–6^ During the poststroke period, myocardial damage could occur as a consequence of 2 opposite forces. First is the activation of neuroinflammatory cascade with the influx of systemic proinflammatory mediators such as IL-1 (interleukin-1) and IL-6, which promotes leukocyte activation and reactive oxygen species production. Second is the immune-suppression activity supported by the anti-inflammatory cytokine IL-10 and antioxidant systems, which limits the extent of immune system activation and prevents the damage to the other tissues.^7,8^

Serum albumin, in addition to its fundamental role in maintaining the oncotic pressure, has important anti-inflammatory properties mainly mediated by its antioxidant activity.^9^ In acute inflammatory conditions, albumin acts as a scavenger that reduces reactive oxygen species bioavailability and when its levels decrease, an impairment of the total plasma antioxidant activity occurs and may promote the onset of cardiovascular events.^10^ In the general population, as well as in patients with cardiovascular disease, albumin levels inversely correlate with the risk of cardiovascular events and death.^11,12^ In 14 506 healthy middle-aged individuals enrolled in the ARIC (Atherosclerosis Risk in Communities) Study, albumin levels were directly correlated with the risk of incident coronary heart disease (hazard ratio [HR], 1.26 [95% CI, 1.15–1.38]).^13^ In 8750 patients with acute myocardial infarction, albumin levels <3.4 g/dL were associated with a higher 10-year risk of all-cause death (HR, 1.70 [95% CI, 1.48–1.95]).^14^ Whereas, in patients with stroke, albumin levels negatively correlate with stroke severity, degree of disability, and functional outcomes.^15–17^ However, no studies have investigated the potential association of albumin levels with the cardiovascular complications of the Stroke Heart Syndrome.

The aim of this study was to evaluate the associations between albumin levels during the first 24 hours after an ischemic stroke and the 30-day risk of cardiovascular events or death in a large cohort of patients with stroke from a federated health research network.

## METHODS

### Data Availability Statement and Ethical Approval

TriNetx is a research network compliant with the Health Insurance Portability and Accountability Act and the US federal law which protects the privacy and security of health care data, including de-identified data as per the deidentification standard of the HIPAA Privacy Rule ([Health Insurance Portability and Accountability Act]; https://trinetx.com/real-world-resources/publications/). The TriNetX research network is utilized for several scientific purposes and to gain access to the data, a sharing agreement is required. As a federated research network, studies using the TriNetX health research network do not need ethical approval as no patient-identifiable identification is received. Further information about the data extraction from TriNetX is reported in the Supplemental Material.

### Study Design

This was a retrospective observational study. TriNetX is a global federated health research network with access to electronic medical records from participating health care organizations including academic medical centers and community hospitals covering ≈80 million individuals, mainly in the United States. Within this network, available data include demographics, diagnoses using *International Classification of Diseases, Tenth Revision, Clinical Modification* codes, measurements (coded to logical observation identifiers names and codes), medications, and medications anatomic Therapeutic Chemical code). The TriNetX database performs internal and extensive data quality assessment with every refresh based on conformance, completeness, and plausibility.^18^ More information can be found in the Supplemental Material. This article follows the STROBE (Strengthening the Reporting of Observational Studies in Epidemiology) reporting guideline.^19^

### Cohort

The searches on the TriNetX online research platform were performed on June 7, 2023 for individuals aged ≥18 years with primary (hospital) discharge *International Classification of Diseases, Tenth Revision, Clinical Modification* codes for ischemic stroke (termed as cerebral infarction: I63) and albumin measurement during the first 24 hours from the admission to hospital, recorded in electronic medical records. To include the highest number of patients possible, the searches were not restricted to a specific period; however, >95% of patients considered in this study were entered into the TriNetX platform between 2000 and 2023. The choice to consider the first 24 hours after admission was made to evaluate the prognostic role of albumin during the acute ischemic stroke phase. At the time of the search, 80 participating health care organizations had data available for patients who met the study inclusion criteria. The baseline index event date was the date of the reported diagnosis of ischemic stroke on the TriNetX platform; any diagnoses registered before this date were considered as the individual’s baseline characteristics.

The cohort was divided into groups using electronic health records according to albumin levels during the first 24 hours after the ischemic stroke. Given the presence of previous studies demonstrated that albumin levels ≤3.4 g/dL are associated with an increased 1-year risk of cardiovascular events in acutely ill medical patients^12,20^ we utilized the same validated cutoff to subdivide patients with stroke into 2 groups: (1) those with normal albumin levels ≥3.5 g/dL, and (2) those with hypoalbuminemia ≤3.4 g/dL.

### Outcomes

The primary outcome was the risk of cardiovascular events within 30 days after the ischemic stroke. Cardiovascular events were defined as a composite outcome of all-cause death, acute myocardial infarction (*International Classification of Diseases, Tenth Revision, Clinical Modification I21*), heart failure (I59), atrial fibrillation and flutter (I48), ventricular arrhythmias (I47.2: ventricular tachycardia and I49.0 ventricular fibrillation), and Takotsubo cardiomyopathy (I51.81) within 30 days after the ischemic stroke. The secondary outcomes were the risk for each component of the composite primary outcome. The occurrence of the primary and secondary outcomes was analyzed based on the levels of albumin measured within the first 24 hours after the index ischemic stroke. The cardiovascular complications of interest within 30 days of ischemic stroke, were identified via *International Classification of Diseases, Tenth Revision, Clinical Modification* code (Table S1).

To estimate the risk of a new cardiovascular complications poststroke, we performed an exploratory analysis excluding patients who experienced the outcome of interest, before the index stroke.

Albumin levels can be influenced by several physiological and pathological conditions. Hence, we decided to perform 5 separate sensitivity analyses to test the reproducibility of the results obtained from the main analysis.

The first sensitivity analysis included patients with age >65 years only. Albumin levels were reported to be lower in older compared to young people.^21^

The second sensitivity analysis included females only. The association between cardiovascular events and albumin levels is less clear in females than in males.^22^

The third sensitivity analysis was done in patients with hypertension and at least on the following comorbidities diabetes, chronic kidney disease, dyslipidemia, heart failure, or previous ischemic heart disease. The association between albumin and cardiovascular events could be less evident in those with multimorbidity at higher risk per se.

The fourth sensitivity analysis was performed subdividing patients with reduced albumin into mild reduced (2.8–3.5 g/dL) and severe reduced (≤2.7 g/dL), as previously described.^23^ This analysis was done to evaluate if a gradient effect between albumin levels and cardiovascular risk can be found.

The last sensitivity analysis included patients with neither cirrhosis, malnutrition, nephrotic syndrome, inflammatory bowel disease, nor autoimmune diseases, who had a normal albumin level (≥3.5 g/dL) 1 month before the index stroke, in the absence of sepsis, burden injury, and trauma. The latter analysis was done to exclude that in some patients the albumin levels were already reduced before the index stroke.

### Statistical Analysis

All statistical analyses were performed on the TriNetX online research platform. Baseline characteristics were compared using χ^2^ tests for categorical variables and independent-sample *t* tests for continuous variables. To create balanced cohorts, we performed a propensity score matching analysis (PSM), using logistic regression. We performed a 1:1 greedy nearest neighbor matching model. Any baseline characteristic with an absolute standardized mean difference between cohorts lower than 0.1 was considered well-matched. We included the following variables in the PSM: age, sex, ethnicity, hypertension, ischemic heart diseases, ischemic stroke, heart failure, pulmonary embolism, diabetes, peripheral arterial disease, pneumonia, sepsis, systemic connective tissue disease (vasculitis, systemic lupus erythematosus, dermatopolyositis, systemic sclerosis, Sjögren syndrome, Behçet disease, polymyalgia rheumatica, multisystem inflammatory syndrome), malnutrition, nephrotic syndrome, liver cirrhosis, inflammatory bowel disease (Crohn disease and ulcerative colitis), burns of external body surface, cardiovascular procedures (including electrocardiography, echocardiography, catheterization procedures), and cardiovascular medications (including anticoagulants, antiplatelets, β-blockers, antiarrhythmics, diuretics, antilipemic agents, antianginals, calcium channel blockers, and angiotensin-converting enzyme inhibitors and angiotensin II receptor blockers). Cox proportional hazard regression was used to compare the matched cohorts. HRs and 95% CIs were calculated to investigate the risk of early cardiovascular complications and all-cause death between stroke patients with reduced and normal albumin levels. Kaplan-Meier curves and log-rank test were used to compare the survival distributions during the follow-up period. Patients were censored when they no longer provided additional information for the analysis. The proportional hazard assumption was tested based on the scaled Schoenfeld residual. All tests were 2-tailed and *P* values of ≤0.05 were taken to indicate statistical significance. All analyses were performed in the TriNetX platform which uses R’s survival package v3.2 to 3.

## RESULTS

**Figure 1.**
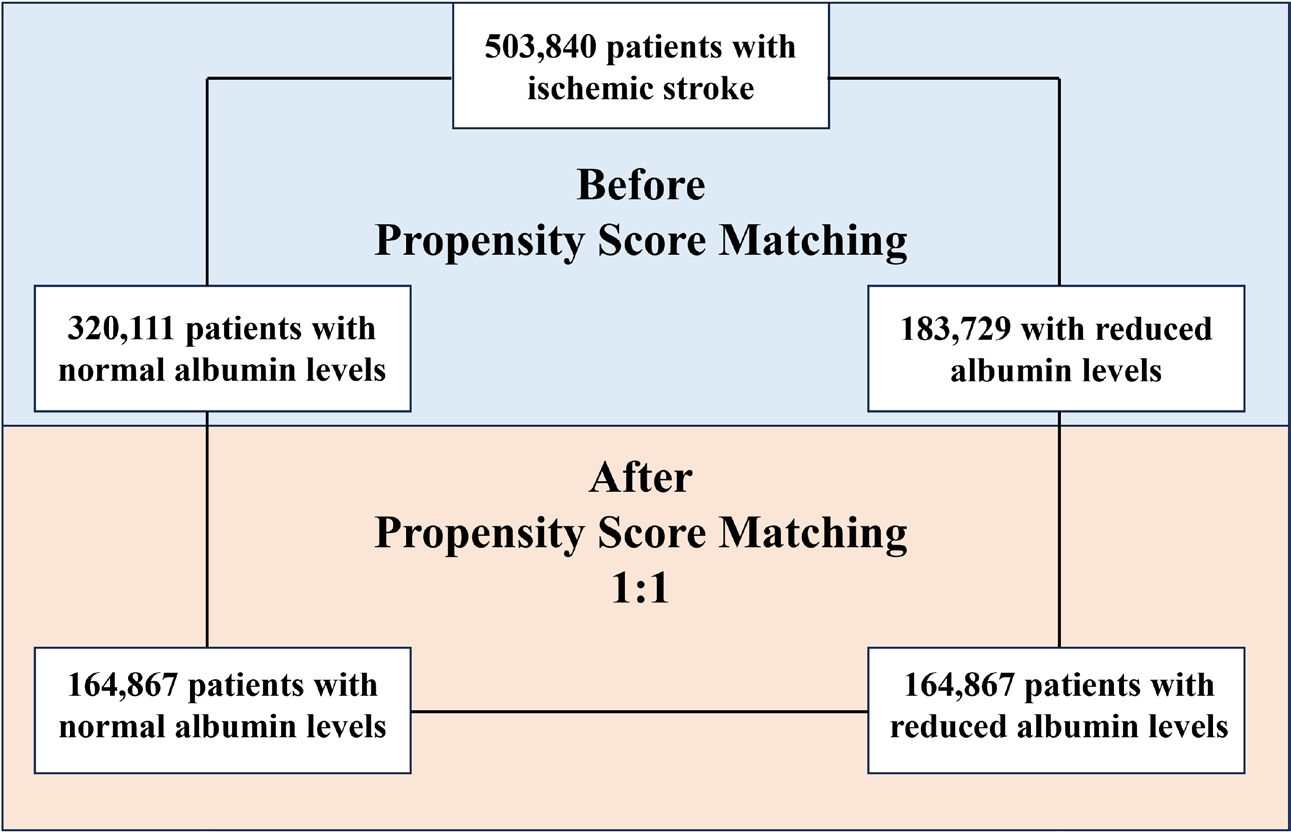
Patients’ flow of the study.

The initial cohorts consisted of 503 840 patients with ischemic stroke, of whom 320 111 with normal albumin levels ≥3.5 g/dL (mean age, 70.9±14.7 years; 48.9% females) and 183 729 with reduced albumin levels ≤3.4 g/dL (mean age, 72.9±14.3 years; 50.3% females; Figure 1).

Before PSM, patients with reduced albumin levels showed a higher prevalence of cardiovascular risk factors, liver cirrhosis, nephrotic syndrome, inflammatory bowel disease, infectious and autoimmune diseases, compared to patients with normal albumin levels (Table 1). After PSM were selected, 164 867 patients for each group and no significative differences were found for all the variables considered (Figure 1; Table 1). Overall were excluded 49.5% of patients with normal albumin levels and 11.3% of patients with reduced albumin levels.

**Table 1.**
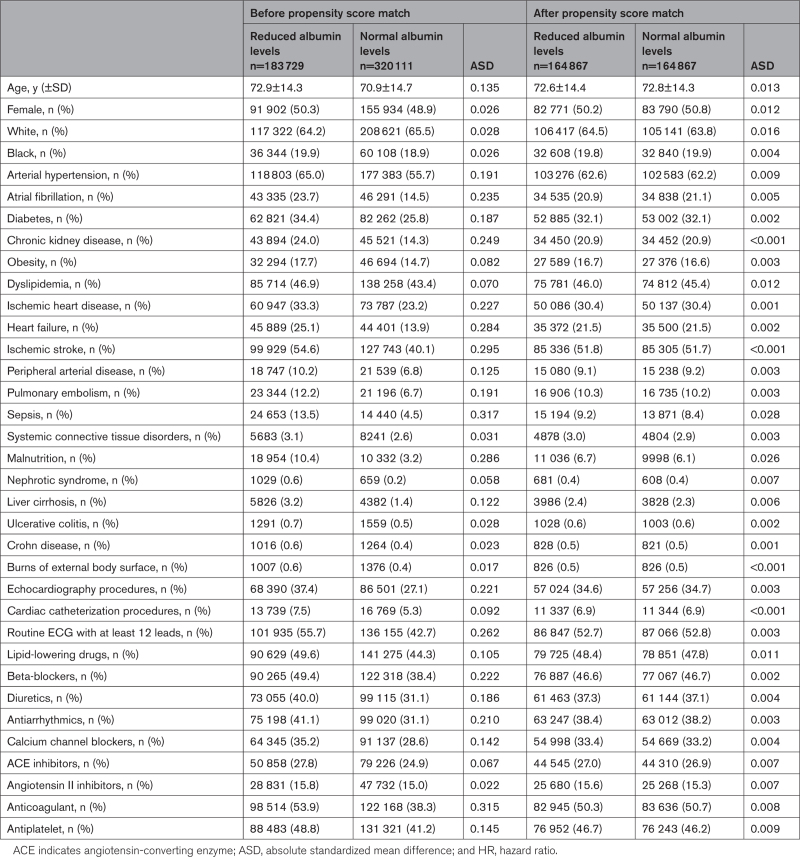
Baseline Characteristics of Patients With Stroke With Reduced and Normal Albumin Levels After Propensity Score Matching

### Risk of Early Cardiovascular Events in Patients With Reduced Albumin Levels

**Figure 2.**
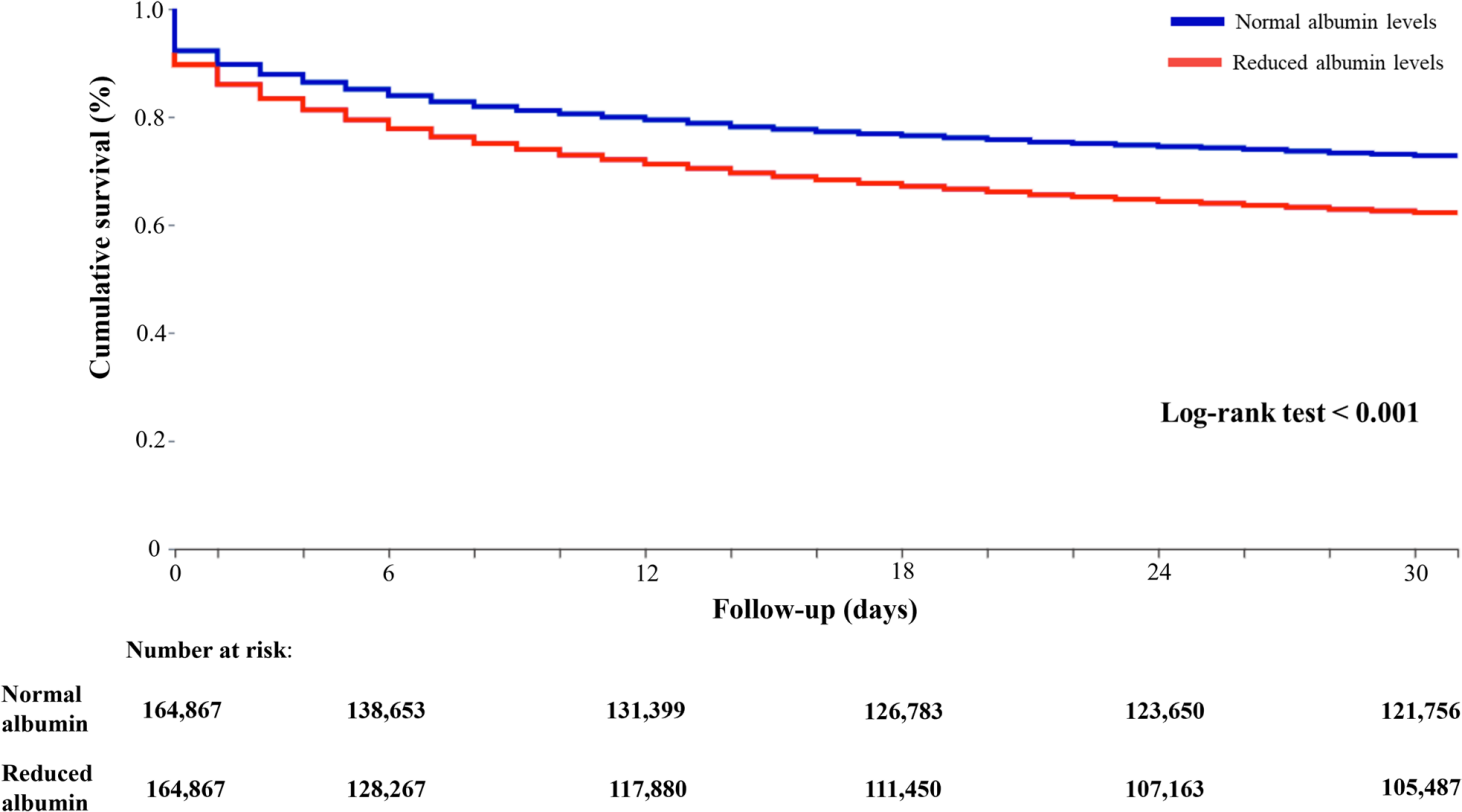
Kaplan-Meier curves for the composite outcome in patients with ischemic stroke based on albumin levels.

After PSM, in the comparisons between patients with reduced and those with normal albumin levels, the primary composite outcome of death and cardiovascular events within 30 days after ischemic stroke was 59 380 (36.0%) and 43 111 (26.1%), respectively (HR, 1.48 [95% CI, 1.46–1.50]; Table 2; Figure 2).

**Table 2.**
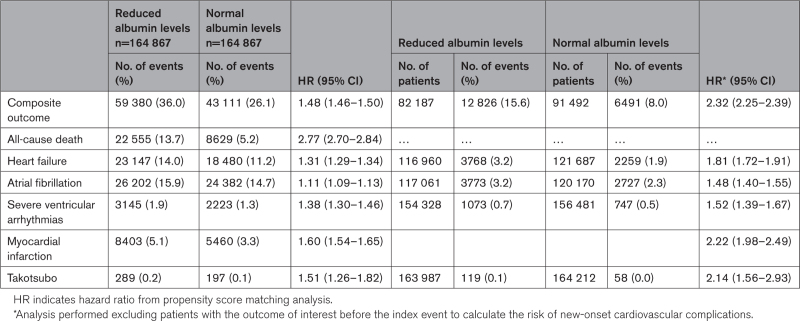
Thirty-Day Risk of Early Cardiovascular Complications After Stroke Based on Albumin Levels

For secondary outcomes, the total number of patients with reduced and normal albumin levels who experienced cardiovascular events were respectively: 22 555 and 8629 all-cause death (HR, 2.77 [95% CI, 2.70–2.84]), 23 147 and 18 480 heart failure (HR, 1.31 [95% CI, 1.29–1.34]), 26 202 and 24 382 atrial fibrillation (HR, 1.11 [95% CI, 1.09–1.13]), 3145 and 2223 severe ventricular arrhythmias (HR, 1.38 [95% CI, 1.30–1.46]), 8403 and 5460 myocardial infarction (HR, 1.60 [95% CI, 1.54–1.65]), and 289 and 197 Takotsubo cardiomyopathy (HR, 1.51 [95% CI, 1.26–1.82]; Table 2).

### Risk of New-Onset Cardiovascular Events in Patients With Reduced Albumin Levels

To estimate the risk of new-onset cardiovascular complications, we performed an exploratory analysis excluding patients who experienced the outcomes of interest before the index stroke.

We found that the composite outcome occurred in 12 826 (15.2%) patients with reduced albumin levels and 6491 (7.1%) patients with normal albumin levels (HR, 2.32 [95% CI, 2.25–2.39]). Consistent with the main analysis, patients with reduced albumin levels showed a higher risk of heart failure (HR, 1.81 [95% CI, 1.72–1.91]), atrial fibrillation (HR, 1.48 [95% CI, 1.40–1.55]), severe ventricular arrhythmias (HR, 1.52 [95% CI, 1.39–1.67]), myocardial infarction (HR, 2.22 [95% CI, 1.98–2.49]), and Takotsubo cardiomyopathy (HR, 2.14 [95% CI, 1.56–2.93]) compared to patients with normal albumin levels (Table 2).

### Sensitivity Analysis

In patients aged >65 years, after PSM, patients with reduced albumin levels were at higher risk of composite outcome (HR, 1.42 [95% CI, 1.40–1.44]), all-cause death (HR, 2.62 [95% CI, 2.55–2.69]), heart failure (HR, 1.29 [95% CI, 1.26–1.32]), atrial fibrillation (HR, 1.11 [95% CI, 1.09–1.13]), severe ventricular arrhythmias (HR, 1.36 [95% CI, 1.28–1.45]), myocardial infarction (HR, 1.56 [95% CI, 1.50–1.62]), and Takotsubo cardiomyopathy (HR, 1.37 [95% CI, 1.10–1.71]), compared to older patients with normal albumin levels (Table S3).

With the analysis performed only in females, after PSM, reduced albumin levels were still associated with a higher risk of composite outcome (HR, 1.47 [95% CI, 1.44–1.50]), all-cause death (HR, 2.64 [95% CI, 2.54–2.73]), heart failure (HR, 1.33 [95% CI, 1.29–1.37]), atrial fibrillation (HR, 1.11 [95% CI, 1.09–1.14]), severe ventricular arrhythmias (HR, 1.51 [95% CI, 1.38–1.66]), myocardial infarction (HR, 1.60 [95% CI, 1.52–1.69]), and Takotsubo cardiomyopathy (HR, 1.64 [95% CI, 1.33–2.03]), compared to female patients with normal albumin levels (Table S3).

After PSM, in 56 960 patients with stroke with multimorbidity for each group, the higher risk of composite outcome (HR, 1.47 [95% CI, 1.44–1.50]), all-cause death (HR, 2.64 [95% CI, 2.54–2.73]), heart failure (HR, 1.33 [95% CI, 1.29–1.37]), atrial fibrillation (HR, 1.11 [95% CI, 1.09–1.14]), severe ventricular arrhythmias (HR, 1.51 [95% CI, 1.38–1.66]), myocardial infarction (HR, 1.60 [95% CI, 1.52–1.69]), and Takotsubo cardiomyopathy (HR, 1.64 [95% CI, 1.33–2.03]) in patients with reduced albumin levels was consistent with the main analysis (Table S3).

When analyzing patients with mild or severe reduced albumin levels, compared to those with normal albumin levels, we found a progressively increased risk of composite outcome (HR, 1.37 [95% CI, 1.35–1.39] and HR, 1.70 [95% CI, 1.67–1.73]), all-cause death (HR, 2.31 [95% CI, 2.25–2.38] and HR, 4.17 [95% CI, 4.01–4.32]), ventricular arrhythmias (HR, 1.38 [95% CI, 1.30–1.57] and HR, 1.54 [95% CI, 1.42–1.66]), myocardial infarction (HR, 1.51 [95% CI, 1.46–1.57] and HR, 1.76 [95% CI, 1.68–1.86]) and Takotsubo (HR, 1.54 [95% CI, 1.26–1.89], and HR, 1.97 [95% CI, 1.51–2.57]), whereas the risk of atrial fibrillation (HR, 1.11 [95% CI, 1.09–1.13] and HR, 1.12 [95% CI, 1.09–1.15]) and heart failure (HR, 1.28 [95% CI, 1.25–1.31] and HR, 1.29 [95% CI, 1.25–1.32]) were substantially the same (Table S3).

**Figure 3.**
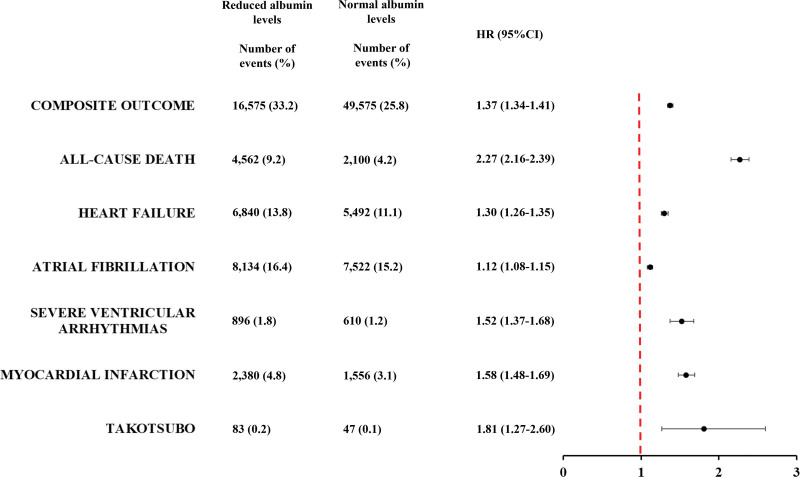
**Thirty-day risk of early cardiovascular complications after stroke based on albumin levels in patients without other causes of hypoalbuminemia.** HR indicates hazard ratio.

Finally, on the analysis performed only in patients with normal albumin levels 1 month before the index stroke, after PSM (Table S2), we found that patients with acute reduced albumin levels were still associated with a higher risk of primary and secondary outcomes, compared to those with normal albumin levels (Figure 3).

## DISCUSSION

In this large observational cohort derived from a global multinational health care network, we showed that albumin levels measured within 24 hours after ischemic stroke were associated with the risk of early cardiovascular events and death, independent of advanced age, female sex, and possible causes of chronic hypoalbuminemia.

Several studies have demonstrated that in the general population albumin levels are inversely related to the incidence risks of coronary artery disease,^13,24^ heart failure,^25,26^ atrial fibrillation,^27^ stroke,^28^ and venous thromboembolic events.^29^ Furthermore, this association was also confirmed in patients with previous cardiovascular events, whereby albumin levels predicted the long- and short-term risks of adverse outcomes.^30–32^ In 15 511 hospitalized patients, aged 40 years or less, albumin levels ≤3.4 g/dL were associated with a high risk of intrahospital death and a high readmission rate within 1 year.^20^ In a recent study on 4152 hospitalized acutely ill medical patients from the REPOSI study (REgistro POliterapie SIMI, Societa Italiana di Medicina Interna), albumin levels ≤3.4 g/dL were associated with a higher 1-year risk of all-cause death (HR, 2.33 [95% CI, 1.93–2.80]) and thrombotic events (HR, 2.16 [95% CI, 1.40–3.31]).^12^ While in patients with stroke, reduced albumin levels have been associated with specific etiologies (cryptogenic and cardioembolic),^28^ and with a higher risk of recurrences,^17^ disability, and death.^15,33^

The possible explanations behind the relationship between reduced albumin levels and the high risk of cardiovascular events are complex and involve different mechanisms. Albumin is a nonglycosylated protein of 66 kDa, which represent up to 60% of total plasma proteins, with important antioxidant, antiplatelet, and anticoagulant effects.^34^ Indeed, albumin acts as reactive oxygen species scavenger reducing the oxidative stress, inactivates thromboxane A_2_ preventing platelet aggregation, and binds antithrombin and factor X contrasting the coagulation activation.^9,35,36^

During the acute stroke phase, albumin represents one of the most important systems that counteract the detrimental effect of the inflammatory response associated with neuronal injury. However, the persistence of this inflammatory state may reduce serum albumin levels leading to an enhanced oxidative stress responsible for endothelial dysfunction, platelet aggregation, and clot initiation. In our study, we found that not only the risk of myocardial infarction but also the risks of heart failure, arrhythmias, and takotsubo cardiomyopathy were increased in patients with reduced albumin levels. Indeed, the increased oxidative stress is not only responsible for thrombotic events but can also lead to myocardial depressed function, arrhythmogenesis, and vasospasm with possible nonthrombotic ischemic manifestations.^2^ Thus, underlying that inflammation and oxidative stress represent a common pathophysiological mechanism that links several cardiovascular diseases.

The antioxidant properties of albumin and its close relationship with the risk of cardiovascular events generates the hypothesis of the therapeutic use of albumin supplementation in Stroke Heart Syndrome. The previous studies that have investigated the potential beneficial effect of albumin infusion in patients with acute stroke have returned conflicting results. A meta-analysis of 1611 patients from 4 different studies found no beneficial effect on the long-term neurological function of patients with ischemic stroke (odds ratio, 1.04 [95% CI, 0.85–1.27]) treated with albumin compared to control.^37^ However, the different albumin dosages, the presence of important bias, and the lack of laboratory experiments that investigated the possible antioxidant or anticoagulant effect of albumin making it difficult to draw unequivocal conclusions.^38–40^ Hence, the dosage of albumin associated with a significant antioxidant or anticoagulant effect should be assessed in advance, and more data are needed to clarify if albumin supplementation could represent a putative therapeutic approach to counteract the poststroke myocardial damage mediated by inflammation and oxidative stress. Nevertheless, the oncotic properties of albumin could represent a possible contraindication in patients with preexisting cardiovascular diseases, and the possible pro-oxidant albumin effect mediated by redox-cycling iron may induce a paradoxical enhanced oxidative stress^41^ that could further worsen the inflammatory poststroke status.

Anyway, beyond these possible therapeutical aspects, this study first reported that albumin levels measured during the 24 hours after ischemic stroke may help to stratify the short-term risk of cardiovascular complications and to identify patients who can benefit from stricter cardiovascular monitoring.

### Limitations

Several limitations should be acknowledged when interpreting these results. Caution is needed in drawing causal conclusions given the retrospective and observational nature of this study that is associated with unmeasured bias. TriNetX is an online research platform that is potentially subjected to entry errors and data gaps. We cannot establish if the outcomes happened after discharge and those that occurred outside the network may have not been well captured. We were not able to investigate the impact or match the included patients based on stroke severity using the National Institutes of Health Stroke Scale or infarct volume since these data were not available in the TriNetX platform. Moreover, we are not able to balance cohorts based on revascularization therapies (ie, intravenous thrombolysis and endovascular thrombectomy), hemorrhagic transformation, or any discharge data, because the TriNetX database does not allow us to balance cohorts based on characteristics recorded after the index event. The albumin levels collected in the electronic database were exhaustive and this may influence other bias impacted our results. We did not consider in the PSM the presence of other possible confounding factors such as the socioeconomic status, quality of care, and risk factor control. Although we adjusted the PSM for several factors involved in determining serum albumin levels, we could not exclude that some clinical conditions that are associated with hypoalbuminemia are associated with a higher risk of cardiovascular events per se. However, in the sensitivity analysis performed excluding other possible causes of hypoalbuminemia, the risk of early cardiovascular complications in patients with stroke was still associated with albumin levels.

### Conclusions

Albumin levels, measured within the first 24 hours after ischemic stroke, are associated with a higher risk of early cardiovascular events and death. The potential pathophysiological or therapeutic roles of albumin in Stroke Heart Syndrome warrants further investigation.

## ARTICLE INFORMATION

### Sources of Funding

None.

### Disclosures

Dr Lip is a consultant and speaker for Bristol-Myers Squibb/Pfizer, Boehringer Ingelheim, Anthos and Daiichi-Sankyo. No fees are received personally. Dr Lip is co-principal investigator of the AFFIRMO project (Atrial Fibrillation Integrated Approach in Frail, Multimorbid and Polymedicated Older People) on multimorbidity in atrial fibrillation, which has received funding from the European Union’s Horizon 2020 research and innovation program under grant agreement no. 899871. The other authors report no conflicts.

### Supplemental Material

Tables S1–S3

STROBE Checklist

## Supplementary Material

## References

[1] Buckley BJR Harrison SL Hill A Underhill P Lane DA Lip GYH. Stroke-heart syndrome: incidence and clinical outcomes of cardiac complications following stroke. Stroke. 2022;53:1759–1763. doi: 10.1161/STROKEAHA.121.037316 35354300 10.1161/STROKEAHA.121.037316

[2] Sposato LA Hilz MJ Aspberg S Murthy SB Bahit MC Hsieh CY Sheppard MN Scheitz JF World Stroke Organisation B Heart Task F. Post-stroke cardiovascular complications and neurogenic cardiac injury: JACC state-of-the-art review. J Am Coll Cardiol. 2020;76:2768–2785. doi: 10.1016/j.jacc.2020.10.009 33272372 10.1016/j.jacc.2020.10.009

[3] Scheitz JF Nolte CH Doehner W Hachinski V Endres M. Stroke-heart syndrome: clinical presentation and underlying mechanisms. Lancet Neurol. 2018;17:1109–1120. doi: 10.1016/S1474-4422(18)30336-3 30509695 10.1016/S1474-4422(18)30336-3

[4] Bucci T Sagris D Harrison SL Underhill P Pastori D Ntaios G McDowell G Buckley BJR Lip GYH. C-reactive protein levels are associated with early cardiac complications or death in patients with acute ischemic stroke: a propensity-matched analysis of a global federated health from the TriNetX network. Intern Emerg Med. 2023;18:1329–1336. doi: 10.1007/s11739-023-03280-1 37119383 10.1007/s11739-023-03280-1 PMC10412660

[5] Ormstad H Aass HC Lund-Sorensen N Amthor KF Sandvik L. Serum levels of cytokines and C-reactive protein in acute ischemic stroke patients, and their relationship to stroke lateralization, type, and infarct volume. J Neurol. 2011;258:677–685. doi: 10.1007/s00415-011-6006-0 21424610 10.1007/s00415-011-6006-0 PMC3065641

[6] Di Napoli M Papa F Bocola V. Prognostic influence of increased C-reactive protein and fibrinogen levels in ischemic stroke. Stroke. 2001;32:133–138. doi: 10.1161/01.str.32.1.133 11136928 10.1161/01.str.32.1.133

[7] Tahsili-Fahadan P Geocadin RG. Heart-brain axis: effects of neurologic injury on cardiovascular function. Circ Res. 2017;120:559–572. doi: 10.1161/CIRCRESAHA.116.308446 28154104 10.1161/CIRCRESAHA.116.308446

[8] McCombe PA Read SJ. Immune and inflammatory responses to stroke: good or bad? Int J Stroke. 2008;3:254–265. doi: 10.1111/j.1747-4949.2008.00222.x 18811742 10.1111/j.1747-4949.2008.00222.x

[9] Taverna M Marie AL Mira JP Guidet B. Specific antioxidant properties of human serum albumin. Ann Intensive Care. 2013;3:4. doi: 10.1186/2110-5820-3-4 23414610 10.1186/2110-5820-3-4 PMC3577569

[10] Arques S. Human serum albumin in cardiovascular diseases. Eur J Intern Med. 2018;52:8–12. doi: 10.1016/j.ejim.2018.04.014 29680174 10.1016/j.ejim.2018.04.014

[11] Pignatelli P Farcomeni A Menichelli D Pastori D Violi F. Serum albumin and risk of cardiovascular events in primary and secondary prevention: a systematic review of observational studies and Bayesian meta-regression analysis. Intern Emerg Med. 2020;15:135–143. doi: 10.1007/s11739-019-02204-2 31605272 10.1007/s11739-019-02204-2

[12] Violi F Novella A Pignatelli P Castellani V Tettamanti M Mannucci PM Nobili A Group RS. Low serum albumin is associated with mortality and arterial and venous ischemic events in acutely ill medical patients. Results of a retrospective observational study. Thromb Res. 2023;225:1–10. doi: 10.1016/j.thromres.2023.02.013 36898171 10.1016/j.thromres.2023.02.013

[13] Nelson JJ Liao D Sharrett AR Folsom AR Chambless LE Shahar E Szklo M Eckfeldt J Heiss G. Serum albumin level as a predictor of incident coronary heart disease: the Atherosclerosis Risk in Communities (ARIC) study. Am J Epidemiol. 2000;151:468–477. doi: 10.1093/oxfordjournals.aje.a010232 10707915 10.1093/oxfordjournals.aje.a010232

[14] Plakht Y Gilutz H Shiyovich A. Decreased admission serum albumin level is an independent predictor of long-term mortality in hospital survivors of acute myocardial infarction. Soroka Acute Myocardial Infarction II (SAMI-II) project. Int J Cardiol. 2016;219:20–24. doi: 10.1016/j.ijcard.2016.05.067 27257851 10.1016/j.ijcard.2016.05.067

[15] Zhou H Wang A Meng X Lin J Jiang Y Jing J Zuo Y Wang Y Zhao X Li H. Low serum albumin levels predict poor outcome in patients with acute ischaemic stroke or transient ischaemic attack. Stroke Vasc Neurol. 2021;6:458–466. doi: 10.1136/svn-2020-000676 33632730 10.1136/svn-2020-000676 PMC8485231

[16] Seet RC Lim EC Chan BP Ong BK. Serum albumin level as a predictor of ischemic stroke outcome. Stroke. 2004;35;2435–2436. doi: 10.1161/01.STR.0000145487.89910.12 15472082 10.1161/01.STR.0000145487.89910.12

[17] Zhang Q Lei YX Wang Q Jin YP Fu RL Geng HH Huang LL Wang XX Wang PX. Serum albumin level is associated with the recurrence of acute ischemic stroke. Am J Emerg Med. 2016;34:1812–1816. doi: 10.1016/j.ajem.2016.06.049 27364646 10.1016/j.ajem.2016.06.049

[18] Kahn MG Callahan TJ Barnard J Bauck AE Brown J Davidson BN Estiri H Goerg C Holve E Johnson SG. A harmonized data quality assessment terminology and framework for the secondary use of electronic health record data. EGEMS (Wash DC). 2016;4:1244. doi: 10.13063/2327-9214.1244 27713905 10.13063/2327-9214.1244 PMC5051581

[19] Vandenbroucke JP von Elm E Altman DG Gotzsche PC Mulrow CD Pocock SJ Poole C Schlesselman JJ Egger M initiative S. Strengthening the Reporting of Observational Studies in Epidemiology (STROBE): explanation and elaboration. Ann Intern Med. 2007;147:W163–W194. doi: 10.7326/0003-4819-147-8-200710160-00010-w1 17938389 10.7326/0003-4819-147-8-200710160-00010-w1

[20] Herrmann FR Safran C Levkoff SE Minaker KL. Serum albumin level on admission as a predictor of death, length of stay, and readmission. Arch Intern Med. 1992;152:125–130. doi: 10.1001/archinte.1992.00400130135017 1728907

[21] Cabrerizo S Cuadras D Gomez-Busto F Artaza-Artabe I Marin-Ciancas F Malafarina V. Serum albumin and health in older people: review and meta analysis. Maturitas. 2015;81:17–27. doi: 10.1016/j.maturitas.2015.02.009 25782627 10.1016/j.maturitas.2015.02.009

[22] Gillum RF Ingram DD Makuc DM. Relation between serum albumin concentration and stroke incidence and death: the NHANES I epidemiologic follow-up study. Am J Epidemiol. 1994;140:876–888. doi: 10.1093/oxfordjournals.aje.a117176 7977275 10.1093/oxfordjournals.aje.a117176

[23] Child CG Turcotte JG. Surgery and portal hypertension. Major Probl Clin Surg. 1964;1:1–85. 4950264

[24] Djousse L Rothman KJ Cupples LA Levy D Ellison RC. Serum albumin and risk of myocardial infarction and all-cause mortality in the Framingham offspring study. Circulation. 2002;106:2919–2924. doi: 10.1161/01.cir.0000042673.07632.76 12460872 10.1161/01.cir.0000042673.07632.76

[25] Gopal DM Kalogeropoulos AP Georgiopoulou VV Tang WW Methvin A Smith AL Bauer DC Newman AB Kim L Harris TB; Health ABC Study. Serum albumin concentration and heart failure risk the health, aging, and body composition study. Am Heart J. 2010;160:279–285. doi: 10.1016/j.ahj.2010.05.022 20691833 10.1016/j.ahj.2010.05.022 PMC2919495

[26] Filippatos GS Desai RV Ahmed MI Fonarow GC Love TE Aban IB Iskandrian AE Konstam MA Ahmed A. Hypoalbuminaemia and incident heart failure in older adults. Eur J Heart Fail. 2011;13:1078–1086. doi: 10.1093/eurjhf/hfr088 21807662 10.1093/eurjhf/hfr088 PMC3177540

[27] Mukamal KJ Tolstrup JS Friberg J Gronbaek M Jensen G. Fibrinogen and albumin levels and risk of atrial fibrillation in men and women (the Copenhagen City Heart Study). Am J Cardiol. 2006;98:75–81. doi: 10.1016/j.amjcard.2006.01.067 16784925 10.1016/j.amjcard.2006.01.067

[28] Xu WH Dong C Rundek T Elkind MS Sacco RL. Serum albumin levels are associated with cardioembolic and cryptogenic ischemic strokes: Northern Manhattan study. Stroke. 2014;45:973–978. doi: 10.1161/STROKEAHA.113.003835 24549868 10.1161/STROKEAHA.113.003835 PMC3966953

[29] Folsom AR Lutsey PL Heckbert SR Cushman M. Serum albumin and risk of venous thromboembolism. Thromb Haemost. 2010;104:100–104. doi: 10.1160/TH09-12-0856 20390234 10.1160/TH09-12-0856 PMC2902783

[30] Biccire FG Pastori D Tanzilli A Pignatelli P Viceconte N Barilla F Versaci F Gaudio C Violi F Tanzilli G. Low serum albumin levels and in-hospital outcomes in patients with ST segment elevation myocardial infarction. Nutr Metab Cardiovasc Dis. 2021;31:2904–2911. doi: 10.1016/j.numecd.2021.06.003 34344545 10.1016/j.numecd.2021.06.003

[31] Ambrosy AP Vaduganathan M Huffman MD Khan S Kwasny MJ Fought AJ Maggioni AP Swedberg K Konstam MA Zannad F; EVEREST trial investigators. Clinical course and predictive value of liver function tests in patients hospitalized for worsening heart failure with reduced ejection fraction: an analysis of the EVEREST trial. Eur J Heart Fail. 2012;14:302–311. doi: 10.1093/eurjhf/hfs007 22357577 10.1093/eurjhf/hfs007

[32] Schillinger M Exner M Mlekusch W Amighi J Sabeti S Schlager O Wagner O Minar E. Serum albumin predicts cardiac adverse events in patients with advanced atherosclerosis - interrelation with traditional cardiovascular risk factors. Thromb Haemost. 2004;91:610–618. doi: 10.1160/TH03-08-0504 14983239 10.1160/TH03-08-0504

[33] Dziedzic T Slowik A Szczudlik A. Serum albumin level as a predictor of ischemic stroke outcome. Stroke. 2004;35:e156–e158. doi: 10.1161/01.STR.0000126609.18735.be 15073386 10.1161/01.STR.0000126609.18735.be

[34] Evans TW. Review article: albumin as a drug--biological effects of albumin unrelated to oncotic pressure. Aliment Pharmacol Ther. 2002;16:6–11. doi: 10.1046/j.1365-2036.16.s5.2.x 12423448 10.1046/j.1365-2036.16.s5.2.x

[35] Joorgensen KA Stoffersen E. Heparin like activity of albumin. Thromb Res. 1979;16:569–574. doi: 10.1016/0049-3848(79)90105-1 516011 10.1016/0049-3848(79)90105-1

[36] Maclouf J Kindahl H Granstrom E Samuelsson B. Interactions of prostaglandin H2 and thromboxane A2 with human serum albumin. Eur J Biochem. 1980;109:561–566. doi: 10.1111/j.1432-1033.1980.tb04828.x 7408901 10.1111/j.1432-1033.1980.tb04828.x

[37] Huang Y Xiao Z. Albumin therapy for acute ischemic stroke: a meta-analysis. Neurol Sci. 2021;42:2713–2719. doi: 10.1007/s10072-021-05244-9 33945037 10.1007/s10072-021-05244-9

[38] Goslinga H Eijzenbach V Heuvelmans JH van der Laan de Vries E Melis VM Schmid-Schonbein H Bezemer PD. Custom-tailored hemodilution with albumin and crystalloids in acute ischemic stroke. Stroke. 1992;23:181–188. doi: 10.1161/01.str.23.2.181 1561645 10.1161/01.str.23.2.181

[39] Shin DH Moon GJ Bang OY. Albumin therapy in acute stroke patients. J Neurol. 2007;254:870–878. doi: 10.1007/s00415-006-0456-9 17431702 10.1007/s00415-006-0456-9

[40] Hill MD Martin RH Palesch YY Tamariz D Waldman BD Ryckborst KJ Moy CS Barsan WG Ginsberg MD Investigators A. The albumin in acute stroke part 1 trial: an exploratory efficacy analysis. Stroke. 2011;42:1621–1625. doi: 10.1161/STROKEAHA.110.610980 21546491 10.1161/STROKEAHA.110.610980 PMC3118599

[41] Gutteridge JM Quinlan GJ Evans TW. Transient iron overload with bleomycin detectable iron in the plasma of patients with adult respiratory distress syndrome. Thorax. 1994;49:707–710. doi: 10.1136/thx.49.7.707 7520607 10.1136/thx.49.7.707 PMC475063

