# Supplementary material for: Albumin Levels and Risk of Early Cardiovascular Complications After Ischemic Stroke: A Propensity-Matched Analysis of a Global Federated Health Network
Source: Stroke. 2024 Feb 7;55(3):604–12. doi: 10.1161/STROKEAHA.123.044248 (PMC10896196; doi:10.1161/STROKEAHA.123.044248)
Supplement: Supplementary file 1 [file str-55-604-s001.pdf]

Albumin levels and risk of early cardiovascular complications after ischemic stroke: A propensity-matched analysis of a global federated health network

Tommaso Bucci, Daniele Pastori, Pasquale Pignatelli, George Ntaios, Azmil H. Abdul-Rahim, Francesco Violi & Gregory Y H Lip.

Supplemental Material

The TriNetX data are collected from member healthcare organizations (HCO) and originates from their primary electronic health records (EHR) system. A typical HCO is a large academic health center with data coming from majority of its affiliates. A single HCO frequently has more than one facility, including main and satellite hospitals. The data are stored on the TriNetX database via a physical server at the institution's data centre or a virtual hosted appliance. The TriNetX platform comprises of a series of these appliances connected into a federated network. This network can broadcast queries to each appliance. Results are subsequently collected and aggregated. Once the data are sent to the network, it is mapped to a standard and controlled set of clinical terminologies and undergoes a data quality assessment including 'data cleaning' that rejects records which do not meet the TriNetX quality standards. The TriNetX database performs internal and extensive data quality assessment with every refresh based on conformance, completeness, and plausibility (http://doi.org/10.13063/2327-9214.1244). HIPAA (Health Insurance Portability and Accountability Act) compliance of the clinical patient data is achieved using deidentification. Available data types within the network include demographics, diagnoses (represented by ICD-10-CM codes), procedures (coded in ICD-10-PCS or CPT), and measurements (coded to LOINC). While extensive information is provided about patients' diagnoses and procedures, other variables (such as socioeconomic and lifetime factors are not comprehensively represented). The advantage of electronic health record data over insurance claim data is that both insured and uninsured patients are included. An advantage of electronic health record data over survey data is that the former represents the diagnostic rates in the population presenting to healthcare facilities. This provides an accurate account of the burden of specific diagnoses on healthcare systems. One primary limitation of relying on diagnoses is that they do not account for undiagnosed patients who might have a condition but have not yet received medical support. Another general limitation of electronic health record data is that a patient may be seen in different HCO for different components of their care. If one healthcare organization is not part of the federated network, then part of their medical records may not be available. Using a network of healthcare organizations, rather than a single site, limits this possibility but does not fully remove it. Propensity Score Matched Analyses Using logistic regression [Logistic Regression of the scikit-learn package in Python (version 3.7)], TriNetX performs a 1:1 greedy nearest neighbor matching model, with a caliper of 0.1 pooled standard deviations. To eliminate bias resulting from nearest neighbor algorithms, the orders of rows are randomized. Any baseline characteristic with a standardized mean difference between cohorts lower than 0.1 is deemed well matched (https://www.tandfonline.com/doi/full/10.1080/00273171.2011.568786).

Table S1. ICD-10-CM codes for early cardiovascular complications.

Early cardiovascular complications	ICD-10-CM-codes
All-cause death	• Deceased (variable codified by TriNetX).
Acute myocardial infarction	• I21 Acute myocardial infarction
Atrial fibrillation	• I48 Atrial fibrillation and flutter
Ventricular arrhythmias	• I49.0 Ventricular fibrillation and flutter<br>and/or<br>• I47.2 Ventricular tachycardia
Heart failure	• I50 Heart failure
Takotsubo cardiopathy	• I51.81 Takotsubo syndrome

Table S2. Baseline characteristics, of stroke patients (without other possible causes of hypoalbuminemia) with reduced (cohort 1, n = 49,807) compared to normal (cohort 2, n = 232,965) albumin levels, before and after propensity score matching.

**Demographics**							
Cohort			Mean ± SD	Patients	% of Cohort	P-Value	Std diff.
1<br>2	Age	Age	72.8 +/- 14.2<br>70.8 +/- 14.7	49,807<br>232,965	100%<br>100%	<0.001	0.140
1<br>2	2106-3	White		32,233<br>154,251	64.7%<br>66.2%	<0.001	0.031
1<br>2	F	Female		24,756<br>112,023	49.7%<br>48.1%	<0.001	0.032
1<br>2	2054-5	Black or African American		10,727<br>43,501	21.5%<br>18.7%	<0.001	0.072
1<br>2	2028-9	Asian		1,043<br>5,743	2.1%<br>2.5%	<0.001	0.025
**Diagnosis**							
Cohort			Mean ± SD	Patients	% of Cohort	P-Value	Std diff.
1<br>2	I10-I16	Hypertension		35,542<br>118,228	71.4%<br>50.7%	<0.001	0.432
1<br>2	I20-I25	Ischemic heart diseases		17,166<br>46,435	34.5%<br>19.9%	<0.001	0.331
1<br>2	I48	Atrial fibrillation and flutter		12,050<br>29,033	24.2%<br>12.5%	<0.001	0.307
1<br>2	I50	Heart failure		12,043<br>25,886	24.2%<br>11.1%	<0.001	0.348
1<br>2	I26-I28	Pulmonary embolism		5,327<br>11,274	10.7%<br>4.8%	<0.001	0.220
1<br>2	E78	Dyslipidemia		26,502<br>93,022	53.2%<br>39.9%	<0.001	0.269
1<br>2	E08-E13	Diabetes mellitus		17,792<br>53,805	35.7%<br>23.1%	<0.001	0.280
1<br>2	E65-E68	Obesity		9,362<br>30,455	18.8%<br>13.1%	<0.001	0.157
1<br>2	N18	Chronic kidney disease		11,178<br>26,782	22.4%<br>11.5%	<0.001	0.295
1<br>2	I63	Cerebral infarction		35,986<br>79,321	72.3%<br>34.0%	<0.001	0.829
1<br>2	I73.9	Peripheral arterial disease		4,891<br>12,803	9.8%<br>5.5%	<0.001	0.163
**Procedure**							
Cohort			Mean ± SD	Patients	% of Cohort	P-Value	Std diff.
1<br>2	1013050	Echocardiography Procedures		21,312<br>54,894	42.8%<br>23.6%	<0.001	0.417
1<br>2	1013071	Cardiac Catheterization Procedures		4,050<br>9,981	8.1%<br>4.3%	<0.001	0.160
1<br>2	1013012	Electrocardiogram, routine ECG with at least 12 leads		31,735<br>89,367	63.7%<br>38.4%	<0.001	0.524
**Medication**							
Cohort			Mean ± SD	Patients	% of Cohort	P-Value	Std diff.
1<br>2	CV350	Antilipemic agents		28,950<br>94,734	58.1%<br>40.7%	<0.001	0.355
1<br>2	CV100	Beta blockers/related		26,886<br>79,167	54.0%<br>34.0%	<0.001	0.411
1<br>2	CV300	Antiarrhythmics		21,315<br>62,222	42.8%<br>26.7%	<0.001	0.343
1<br>2	CV700	Diuretics		20,268<br>63,217	40.7%<br>27.1%	<0.001	0.289

Cohort	Code	Characteristic	Mean ± SD	Patients	% of Cohort	P-Value	Std diff.
1<br>2	CV200	Calcium channel blockers		19,633<br>59,085	39.4%<br>25.4%	<0.001	0.304
1<br>2	CV800	ACE inhibitors		15,588<br>52,802	31.3%<br>22.7%	<0.001	0.195
1<br>2	CV805	Angiotensin ii inhibitor		8,971<br>31,912	18.0%<br>13.7%	<0.001	0.118
1<br>2	CV250	Antianginals		9,223<br>26,324	18.5%<br>11.3%	<0.001	0.204
1<br>2	BL110	Anticoagulants		29,474<br>76,908	59.2%<br>33.0%	<0.001	0.544
1<br>2	BL117	Platelet aggregation inhibitors		28,058<br>86,249	56.3%<br>37.0%	<0.001	0.395
**Cohort 1 (N = 49,575) and cohort 2 (N = 49,575) characteristics after propensity score matching**							
**Demographics**							
Cohort			Mean ± SD	Patients	% of Cohort	P-Value	Std diff.
1<br>2	Age	Age	72.8 +/- 14.2<br>72.6 +/- 14.1	49,575<br>49,575	100%<br>100%	0.030	0.014
1<br>2	2106-3	White		32,105<br>31,835	64.8%<br>64.2%	0.073	0.011
1<br>2	F	Female		24,628<br>24,867	49.7%<br>50.2%	0.129	0.010
1<br>2	2054-5	Black or African American		10,651<br>10,802	21.5%<br>21.8%	0.244	0.007
1<br>2	2028-9	Asian		1,037<br>1,009	2.1%<br>2.0%	0.532	0.004
**Diagnosis**							
Cohort			Mean ± SD	Patients	% of Cohort	P-Value	Std diff.
1<br>2	I10-I16	Arterial hypertension		35,323<br>35,986	71.3%<br>72.6%	<0.001	0.030
1<br>2	I20-I25	Ischemic heart diseases		17,002<br>17,229	34.3%<br>34.8%	0.129	0.010
1<br>2	I48	Atrial fibrillation and flutter		11,908<br>11,888	24.0%<br>24.0%	0.882	0.001
1<br>2	I50	Heart failure		11,853<br>11,801	23.9%<br>23.8%	0.698	0.002
1<br>2	I26-I28	Pulmonary embolism		5,233<br>5,056	10.6%<br>10.2%	0.065	0.012
1<br>2	E78	Dyslipidemia		26,397<br>26,930	53.2%<br>54.3%	0.001	0.022
1<br>2	E08-E13	Diabetes mellitus		17,655<br>17,868	35.6%<br>36.0%	0.158	0.009
1<br>2	E65-E68	Obesity		9,308<br>9,462	18.8%<br>19.1%	0.212	0.008
1<br>2	N18	Chronic kidney disease		11,019<br>10,956	22.2%<br>22.1%	0.630	0.003
1<br>2	I63	Cerebral infarction		35,754<br>35,660	72.1%<br>71.9%	0.506	0.004
1<br>2	I73.9	Peripheral arterial disease		4,838<br>4,947	9.8%<br>10.0%	0.246	0.007
**Procedure**							
Cohort			Mean ± SD	Patients	% of Cohort	P-Value	Std diff.
1<br>2	1013050	Echocardiography Procedures		21,128<br>21,650	42.6%<br>43.7%	0.001	0.021
1<br>2	1013071	Cardiac Catheterization Procedures		4,017<br>4,078	8.1%<br>8.2%	0.479	0.004
1<br>2	1013012	Electrocardiogram, routine ECG with at least 12 leads		31,504<br>32,391	63.5%<br>65.3%	<0.001	0.037

Medication							
Cohort			Mean ± SD	Patients	% of Cohort	P-Value	Std diff.
1<br>2	CV350	Antilipemic agents		28,806<br>29,289	58.1%<br>59.1%	0.002	0.020
1<br>2	CV100	Beta blockers/related		26,682<br>27,206	53.8%<br>54.9%	0.001	0.021
1<br>2	CV300	Antiarrhythmics		21,138<br>21,489	42.6%<br>43.3%	0.024	0.014
1<br>2	CV700	Diuretics		20,101<br>20,469	40.5%<br>41.3%	0.017	0.015
1<br>2	CV200	Calcium channel blockers		19,470<br>19,647	39.3%<br>39.6%	0.250	0.007
1<br>2	CV800	ACE inhibitors		15,500<br>15,870	31.3%<br>32.0%	0.012	0.016
1<br>2	CV805	Angiotensin ii inhibitor		8,942<br>9,117	18.0%<br>18.4%	0.150	0.009
1<br>2	CV250	Antianginals		9,153<br>9,365	18.5%<br>18.9%	0.084	0.011
1<br>2	BL110	Anticoagulants		29,247<br>29,917	59.0%<br>60.3%	<0.001	0.028
1<br>2	BL117	Platelet aggregation inhibitors		27,899<br>28,278	56.3%<br>57.0%	0.015	0.015

Table S3. Risk of primary and secondary outcomes in the sensitivity analyses.

	Reduced albumin events, n (%)	Normal albumin events, n (%)	HR (95%CI)
**Patients aged >65 years n = 121,807 (each group)**			
Composite outcome, n (%)	47,157 (38.7)	35,711 (29.3)	1.42 (1.40-1.44)
All-cause death, n (%)	17,711 (14.5)	7,216 (5.9)	2.62 (2.55-2.69)
Heart failure, n (%)	17,846 (14.7)	14,566 (12.0)	1.29 (1.26-1.32)
Atrial fibrillation, n (%)	23,055 (18.9)	21,670 (17.8)	1.11 (1.09-1.13)
Severe ventricular arrhythmias, n (%)	2,194 (1.8)	1,682 (1.4)	1.36 (1.28-1.45)
Myocardial infarction, n (%)	6,243 (5.1)	4,163 (3.4)	1.56 (1.50-1.62)
Takotsubo, n (%)	187 (0.2)	141 (0.1)	1.37 (1.10-1.71)
**Females n = 83,216 (each group)**			
Composite outcome, n (%)	28,515 (34.3)	20,770 (25.0)	1.47 (1.44-1.50)
All-cause death, n (%)	10,815 (13.0)	4,336 (5.2)	2.64 (2.54-2.73)
Heart failure, n (%)	10,938 (13.1)	8,605 (10.3)	1.33 (1.29-1.37)
Atrial fibrillation, n (%)	12,644 (15.2)	11,807 (14.2)	1.11 (1.09-1.14)
Severe ventricular arrhythmias, n (%)	1,107 (1.3)	762 (0.9)	1.51 (1.38-1.66)
Myocardial infarction, n (%)	3,757 (4.5)	2,431 (2.9)	1.60 (1.52-1.69)
Takotsubo, n (%)	224 (0.3)	141 (0.2)	1.64 (1.33-2.03)
**Patients with multimorbidity n = 56,960 (each group)**			
Composite outcome, n (%)	20,038 (35.2)	13,467 (23.6)	1.47 (1.44-1.50)
All-cause death, n (%)	8,845 (15.5)	3,689 (6.5)	2.64 (2.54-2.73)
Heart failure, n (%)	7,092 (12.5)	4,921 (8.6)	1.33 (1.29-1.37)
Atrial fibrillation, n (%)	7,635 (13.4)	6,781 (11.9)	1.11 (1.09-1.14)
Severe ventricular arrhythmias, n (%)	955 (1.7)	663 (1.2)	1.51 (1.38-1.66)
Myocardial infarction, n (%)	3,282 (5.8)	2,043 (3.6)	1.60 (1.52-1.69)
Takotsubo, n (%)	108 (0.2)	88 (0.2)	1.64 (1.33-2.03)
**Mild reduced albumin n = 135,572 (each group)**			
Composite outcome, n (%)	47,124 (34.8)	36,501 (26.9)	1.37 (1.35-1.39)
All-cause death, n (%)	15,429 (11.4)	6,994 (5.2)	2.31 (2.25-2.38)
Heart failure, n (%)	19,702 (14.5)	16,049 (11.8)	1.28 (1.25-1.31)
Atrial fibrillation, n (%)	22,254 (16.4)	20,716 (15.3)	1.11 (1.09-1.13)
Severe ventricular arrhythmias, n (%)	2,613 (1.9)	1,959 (1.4)	1.38 (1.30-1.57)
Myocardial infarction, n (%)	6,784 (5.0)	4,635 (3.4)	1.51 (1.46-1.57)
Takotsubo, n (%)	239 (0.2)	159 (0.1)	1.54 (1.26-1.89)
**Severe reduced albumin n = 56,437 (each group)**			
Composite outcome, n (%)	26,914 (45.3)	17,769 (29.9)	1.70 (1.67-1.73)
All-cause death, n (%)	12,881 (21.7)	3,457 (5.8)	4.17 (4.01-4.32)
Heart failure, n (%)	10,060 (16.9)	8,407 (14.1)	1.29 (1.25-1.32)
Atrial fibrillation, n (%)	10,499 (17.7)	9,972 (16.8)	1.12 (1.09-1.15)
Severe ventricular arrhythmias, n (%)	1,443 (2.4)	1,006 (1.7)	1.54 (1.42-1.66)
Myocardial infarction, n (%)	3,839 (6.5)	2,321 (3.9)	1.76 (1.68-1.86)
Takotsubo, n (%)	153 (0.3)	83 (0.1)	1.97 (1.51-2.57)

HR: Hazard Ratio, CI: Confidence of Interval
